# The Effect of Standardised Flower Extracts of *Sorbus aucuparia* L. on Proinflammatory Enzymes, Multiple Oxidants, and Oxidative/Nitrative Damage of Human Plasma Components In Vitro

**DOI:** 10.1155/2019/9746358

**Published:** 2019-02-04

**Authors:** Monika A. Olszewska, Joanna Kolodziejczyk-Czepas, Magdalena Rutkowska, Anna Magiera, Piotr Michel, Marcin W. Rejman, Pawel Nowak, Aleksandra Owczarek

**Affiliations:** ^1^Department of Pharmacognosy, Faculty of Pharmacy, Medical University of Lodz, 90-151 Lodz, Poland; ^2^Department of General Biochemistry, Faculty of Biology and Environmental Protection, University of Lodz, 90-236 Lodz, Poland

## Abstract

Polyphenol-rich plant extracts might alleviate the negative impact of oxidative stress and inflammation, but careful phytochemical standardisation and evaluation of various mechanisms are required to fully understand their effects. In this context, flower extracts of *Sorbus aucuparia* L.—a traditional medicinal plant—were investigated in the present work. The LC-MS/MS profiling of the extracts, obtained by fractionated extraction, led to the identification of 66 constituents, mostly flavonols (quercetin and sexangularetin glycosides with dominating isoquercitrin), pseudodepsides of quinic and shikimic acids (prevailing isomers of chlorogenic acid and cynarin), and flavanols (catechins and proanthocyanidins). Minor extract components of possible chemotaxonomic value were flavalignans (cinchonain I isomers) and phenylamides (spermidine derivatives). As assessed by HPLC-PDA and UV-spectrophotometric studies, the extracts were polyphenol-abundant, with the contents up to 597.6 mg/g dry weight (dw), 333.9 mg/g dw, 382.0 mg/g dw, and 169.0 mg/g dw of total phenolics, flavonoids, proanthocyanidins, and caffeoylquinic acids, respectively. Their biological *in vitro* effects were phenolic-dependent and the strongest for diethyl ether, ethyl acetate, and *n*-butanol fractions of the methanol-water (7 : 3, *v*/*v*) extract. The extracts showed significant, concentration-dependent ability to scavenge *in vivo*-relevant radical/oxidant agents (O_2_^∙−^, OH^∙^, H_2_O_2_, ONOO^–^, NO^∙^, and HClO) with the strongest effects towards OH^∙^, ONOO^–^, HClO, and O_2_^∙−^ (compared to ascorbic acid). Moreover, the extracts efficiently inhibited lipoxygenase and hyaluronidase (compared to indomethacin) but were inactive towards xanthine oxidase. At *in vivo*-relevant levels (1-5 *μ*g/mL), they also effectively protected human plasma components (proteins and lipids) against ONOO^–^-induced oxidative damage (reduced the levels of 3-nitrotyrosine, lipid hydroperoxides, and thiobarbituric acid-reactive substances) and normalised/enhanced the total nonenzymatic antioxidant capacity of plasma. In cytotoxicity tests, the extracts did not affect the viability of human PBMCs and might be regarded as safe. The results support the application of the extracts in the treatment of oxidative stress-related pathologies cross-linked with inflammatory changes.

## 1. Introduction

The regular intake of plant products rich in polyphenols is associated with the reduced risk of NCDs, including cardiovascular disease, atherosclerosis, age-related neurodegenerative disorders, diabetes, and some types of cancer [1]. In the treatment of NCDs that are often multicausal, combined therapies are usually most efficient, as they offer the advantage of additive and synergistic effects [2]. Complex chemical composition and relevant synergy of polyphenolic extracts are thus probably behind their ability to modulate multiple NCD-related pathologies. A pivotal role in the initiation and progression of NCDs is ascribed to the interdependent processes of oxidative stress and inflammation [3, 4]. As free radical scavengers, metal chelators, inhibitors of proinflammatory enzymes, and modifiers of cell signalling pathways, polyphenols may protect cells against oxidative stress-related damage and support normal cellular metabolism and functions [1]. Apart from their dietary role as constituents of fruits and vegetables, polyphenols attract increased attention as components of standardised plant extracts applicable in the adjuvant therapy of NCDs. Indeed, some selected extracts, for instance, those of hawthorn fruit/flower, grapevine leaf/seeds, olive leaf, and chokeberry fruit, have already been introduced worldwide as antioxidant nutraceuticals, and extensive research is being conducted to broaden the spectrum of the relevant plant-based products [5].

*Sorbus aucuparia* L. (rowan, European mountain ash) is a wild rosaceous tree occurring and cultivated across Europe and Asia [6]. Flowers, leaves, and edible fruits (rowanberries) of *S. aucuparia* are traditionally used for diuretic, antidiabetic, anti-inflammatory, antiatherogenic, vasoprotective, vasorelaxant, and antidiarrheal properties [7, 8]. These activities are commonly linked with polyphenolic components, especially flavonoids (quercetin, kaempferol, and sexangularetin glycosides), anthocyanins (cyanidin glycosides), tannin-type proanthocyanidins, and caffeoylquinic acids (CHA isomers), forming unique and diversified profiles in particular organs and/or plant parts, among which the flowers are the least characterised [9–11]. The accumulating research indicates all rowan tissues as strong antioxidants [9, 11–13] and the flowers as exhibiting the highest total phenolic content (TPC) and superior activity parameters [13]. Our previous screening study revealed that, in terms of TPC values and antioxidant capacity, *S. aucuparia* flowers are in the top five of the twenty-four most ethnobotanically relevant raw materials in the large genus *Sorbus* [14]. Moreover, the TPC levels of the dry extracts of rowan flowers and especially their refined fractions of ethyl acetate and *n*-butanol were comparable with those observed for plant extracts effective in the prevention of oxidative stress-related ailments, such as grape seed, green tea, and green mate [10]. All these activity results, although promising, were obtained in simple chemical tests measuring only basic reducing capacity of the extracts towards transition metal ions and stable synthetic radicals (DPPH, ABTS) of relatively high molecular masses. However, in biological systems, oxidative stress is generated by low-molecular, short-lived ROS such as superoxide (O_2_^∙–^), hydrogen peroxide (H_2_O_2_), hydroxyl radical (HO^∙^), nitric oxide (NO^∙^), peroxynitrite (ONOO^–^), and hypochlorous acid (HClO) [3]. Moreover, the activity of polyphenols in cells, tissues, and body fluids depends on their affinity to proteins, which may significantly influence the bioavailability, antioxidant effectiveness, and interactions of polyphenols (usually inhibition) with enzymatic systems [15]. As some of the proinflammatory enzymes produce ROS or are secreted in response to ROS-dependent stimuli, their inhibition forms the bridge between antioxidant and anti-inflammatory activities of polyphenols [4]. On the other hand, some polyphenols may be cytotoxic towards mammalian cells, mainly by exhibiting prooxidant effects [16].

Therefore, the objective of this study was to verify the antioxidant activity of the flower dry extract of *S. aucuparia* in different *in vitro* models including the chemically based tests towards six radical and nonradical oxidants of physiological significance and the biological model of human plasma exposed to oxidative/nitrative stress generated by ONOO^–^. Moreover, the potential inhibitory activity towards three proinflammatory and prooxidant enzymes (LOX, HYAL, and XO) and cellular safety of the extracts (cytotoxicity against human peripheral blood mononuclear cells) were also evaluated. All activity studies were performed for extracts standardised by comprehensive phytochemical profiling using complementary UHPLC-PDA-ESI-MS^3^, HPLC-PDA, and UV-spectrophotometric methods.

## 2. Materials and Methods

### 2.1. Plant Material and Extract Preparation

Flowers of *Sorbus aucuparia* L. were collected and authenticated in May 2015 in the Arboretum (51°49′N, 19°53′E), Forestry Experimental Station of Warsaw University of Life Science (SGGW) in Rogow (Poland). The raw material was dried under normal conditions, powdered with an electric grinder, and subjected to fractionated extraction as previously described [10] to obtain the basic extract MED and its DEF, EAF, BF, and WR fractions. The organic solvent extracts were evaporated *in vacuo*, and the water-containing fractions were lyophilized using an Alpha 1–2/LD Plus freeze dryer (Christ, Osterode am Harz, Germany) and stored at 4°C. In further analyses, freshly prepared solutions of the extracts in methanol-water (7 : 3, *v/v*) were used. All quantitative results were calculated per extract dw.

### 2.2. Phytochemical Standardisation

The qualitative profiling (UHPLC-PDA-ESI-MS^3^ analysis) of the extracts was performed on a UHPLC-3000 RS system (Dionex, Dreieich, Germany) equipped with a dual low-pressure gradient pump, an autosampler, a column compartment, a diode array detector, and an AmaZon SL ion trap mass spectrometer with an ESI interface (Bruker Daltonik, Bremen, Germany). Separations were carried out on a Kinetex XB-C18 column (1.7 *μ*m, 150 mm × 2.1 mm i.d.; Phenomenex, Torrance, CA, USA) at 25°C. The mobile phase consisted of solvent A (water/acetonitrile/formic acid, 95 : 5 : 0.1, *v/v/v*), and solvent B (acetonitrile/formic acid, 100 : 0.1, *v/v*) with the elution profile as follows: 0–45 min, 6%–26% B (*v/v*); 45–55 min, 26%–95% B; 55–63 min, 95% B; 63–70 min, 95%–6% B; and 70–80 min, 6% B (equilibration). All solvents (Avantor Performance Materials, Gliwice, Poland) were of HPLC-grade purity. The flow rate was 0.3 mL/min. Before injection, sample solutions of the extracts (3.0 mg/mL) were filtered through a PTFE syringe filter (13 mm, 0.2 *μ*m, Whatman, Pittsburgh, PA, USA). UV-Vis spectra were recorded over the range of 200–600 nm. The LC eluate was introduced directly into the ESI interface without splitting and analysed in a negative ion mode. The ESI parameters are as follows: the nebulizer pressure was 40 psi; dry gas flow 9 L/min; dry temperature 300°C; and capillary voltage 4.5 kV. MS*^2^* and MS*^3^* fragmentations were obtained in Auto MS/MS mode for the most abundant ions at the time. Analysis was carried out using scan from *m/z* 200 to 2200.

The total phenolic contents (TPC) and total proanthocyanidin contents (TPA) were quantified by the Folin-Ciocalteu and *n*-butanol-HCl methods, respectively, as described previously [10]. Results were expressed as equivalents of gallic acid (GAE) and cyanidin chloride (CYE), respectively.

The quantitative HPLC-PDA-fingerprint assays were performed according to Olszewska et al. [10] using the same equipment and procedure. The phenolic analytes were quantified as equivalents of HPLC-pure external standards (Sigma-Aldrich, Seelze, Germany/St. Louis, MO, USA): flavan-3-ols, flavalignans, and proanthocyanidins as ECA; hydroxybenzoic acids as protocatechuic or *p*-hydroxybenzoic acids; monocaffeoylquinic acid isomers as CHA; dicaffeoylquinic acid isomers as cynarin; hydroxycinnamic acid derivatives including spermidine isomers as caffeic or *p*-coumaric acids; flavonoid monoglycosides as IQ; flavonoid diglycosides as RT; and flavonoid aglycones as QU, depending on the PDA spectra.

### 2.3. Antioxidant Activity Assays against Multiple Oxidants

The antioxidant activity was evaluated *in vitro* by different spectrophotometric and fluorimetric methods following reported literature and using microplate readers SPECTROstar Nano (BMG Labtech, Ortenberg, Germany) and Synergy HTX (BioTek, Winooski, VT, USA). The scavenging efficacy towards O_2_^∙−^ was evaluated in a xanthine/xanthine oxidase system with nitrotetrazolium blue chloride (NBT) used for detection according to Michel et al. [17]. The ability to scavenge HO^∙^ was assayed by the method of Fu et al. [18] with the level of HO^∙^ (generated in Fenton reaction) monitored in the presence of salicylic acid. The NO^∙^-scavenging activity was evaluated according to Czerwińska et al. [19] using diaminofluorescein-2 as NO^∙^ probe. The reducing activity towards H_2_O_2_ was determined following the method of Banothu et al. [20] through direct measurement of the oxidant's absorbance. The ability to scavenge ONOO^−^ was determined by the measurement of the inhibition of Evans blue dye oxidation according to Krzyzanowska-Kowalczyk et al. [21]. The HClO-scavenging effect was assayed by the method of Czerwińska et al. [19] with 5-thio-2-nitrobenzoic acid used for detection. The results of triplicate determinations were expressed as SC_50_ values (defined as the concentration sufficient to obtain 50% of a maximum scavenging capacity), calculated from concentration-inhibition curves, and recalculated into equivalents of AA and TX. All reagents and standards for the assays were obtained from Sigma-Aldrich (Seelze, Germany/St. Louis, MO, USA).

### 2.4. Antioxidant Activity in Human Plasma Model

#### 2.4.1. Isolation of Blood Plasma and Sample Preparation

Blood from healthy, nonsmoking volunteers declaring balanced diet free of antioxidant supplements was obtained from the Regional Centre of Blood Donation and Blood Treatment in Lodz (Poland), collected on CPD (citrate/phosphate/dextrose) solution in the Fresenius-Kabi Compoflex bags, and plasma was isolated by differential centrifugation of the blood [22]. The study was conducted in accordance with the Declaration of Helsinki and all experiments were approved by the committee on the Ethics of Research at the University of Lodz (8/KBBN-UŁ/II/2015). For the FRAP assay and measurements of 3-NT, plasma samples were diluted with a (Ca^2+^)-free phosphate-buffered saline (PBS; 1 : 4, *v/v*) purchased from Biomed (Lublin, Poland), whereas for LOOH and TBARS assays, plasma was diluted with (Ca^2+^)-free PBS in a volume ratio 1 : 1. All samples were preincubated for 5 min at 37°C with the examined extracts added to the final concentration range of 1-50 *μ*g/mL and then exposed to 150 *μ*M (the FRAP assay) or 100 *μ*M (the remaining experiments on blood plasma) of ONOO^–^. Control samples were prepared with plasma untreated with the extracts and/or ONOO^–^. In the experiments with blood plasma and the extracts only (without adding ONOO^–^), no significant differences (*p* > 0.05) were found between the levels of the tested biomarkers in the plasma samples incubated with the extracts and control (untreated) serum. Protein concentration in blood plasma was estimated using the bicinchoninic acid (BCA) assay with the Pierce BCA Protein Assay Kit (Thermo Scientific, Waltham, MA, USA) according to the manufacturer protocol. ONOO^–^ was synthesised as reported earlier [21]. All tests in blood plasma were conducted using 96-well plates and a microplate reader, SPECTROstar Nano (BMG Labtech, Ortenberg, Germany).

#### 2.4.2. Determination of 3-NT in Plasma Proteins

Detection of 3-NT-containing proteins by the competitive ELISA (C-ELISA) method in plasma samples (control or antioxidants and ONOO^–^-treated plasma) was performed according to Kolodziejczyk-Czepas et al. [22] using immunoreagents purchased from Abcam (Cambridge, UK). The concentrations of nitrated proteins were estimated from the standard curve of nitrated fibrinogen (Fg) and expressed as the 3NT-Fg equivalents (in nmol/mg of plasma protein).

#### 2.4.3. Determination of LOOH in Plasma Lipids

Concentration of hydroperoxides in plasma samples (control or antioxidants and ONOO^–^-treated plasma) was determined by the ferric-xylenol orange (FOX-1) protocol according to Kolodziejczyk-Czepas et al. [22]. The FOX-1 reagent contained 125 *μ*M xylenol orange and 100 mM sorbitol in 25 mM sulphuric acid and was freshly prepared each time before use by the addition of ammonium ferrous sulphate to the final concentration of 250 *μ*M. All reagents were purchased from Sigma-Aldrich (Seelze, Germany/St. Louis, MO, USA). To perform the assay, blood plasma samples were mixed with the reagent in a volume ratio 1 : 9 and incubated for 30 min in the dark (25°C). Absorbance of the samples was measured at 560 nm against blank (water instead of the plasma). The amount of lipid hydroperoxides was calculated from the standard curve of hydrogen peroxide and expressed in nmol/mg of plasma proteins.

#### 2.4.4. TBARS Test

Determination of TBARS in plasma samples (control or antioxidants and ONOO^–^-treated plasma) was performed according to Kolodziejczyk-Czepas et al. [23]. 2-Thiobarbituric acid and other reagents were purchased from Sigma-Aldrich (Seelze, Germany/St. Louis, MO, USA). Results were expressed in *μ*mol TBARS/mL of plasma.

#### 2.4.5. FRAP Assay

The influence of the extracts on the NEAC of plasma was determined according to Kolodziejczyk-Czepas et al. [24] with some modifications. The plasma samples prepared as described above were added to the reagent mixture in a volume ratio of 1 : 10 : 1 : 1 for plasma, acetate buffer (300 mM, pH 3.6), TPTZ (2,4,6-tris-(2-pyridyl)-s-triazine; 10 mM, in 0.04 M hydrochloric acid), and ferric chloride (20 mM), respectively. After incubation for 15 min at 37°C, the measured FRAP of plasma samples (control or antioxidants and ONOO^–^-treated plasma) was expressed in millimolar equivalents of ferrous (Fe^2+^) ions calculated from the calibration curve of ferrous sulphate. All reagents were purchased from Sigma-Aldrich (Seelze, Germany/St. Louis, MO, USA).

### 2.5. Inhibition of Proinflammatory Enzymes

The ability of the extracts to inhibit LOX and HYAL was examined according to Matczak et al. [25]. The inhibitory activity towards XO was analysed according to Michel et al. [17]. All reagents and standards for the study including IND, bovine testis HYAL, LOX from soybean, and XO were purchased from Sigma-Aldrich (Seelze, Germany/St. Louis, MO, USA). Results were expressed as IC_50_ values calculated from concentration-inhibition curves.

### 2.6. Cellular Safety Studies

Cytotoxicity of the examined extracts was determined in an experimental model of PBMCs. Cells were isolated from human blood using the Histopaque®-1077 medium purchased from Sigma-Aldrich (Seelze, Germany/St. Louis, MO, USA) as a sterile solution of polysucrose (57 g/L) and sodium diatrizoate (90 g/L) with a density of 1.077 g/mL. From each of the six donors, two independent PBMC isolations and incubations with the extracts were performed. Blood was carefully layered (in a volume ratio of 1 : 1) onto the medium and centrifuged for 30 min (400×*g*, at room temperature). Then, the pellet was washed two times with 0.02 M PBS buffer. The obtained fraction of PBMCs was suspended in PBS. Cell suspensions (1 × 10^6^ PBMCs/mL) were incubated for 1 and 2 h with plant extracts added to the final concentration of 5 and 50 *μ*g/mL (at 37°C). Cell viability (%) was determined during a spectrofluorimetric analysis, involving the use of propidium iodide as a fluorescent dye. Measurements were conducted using a microchip-type automatic cell counter Adam-MC DigitalBio (NanoEnTek Inc., Seoul, Korea) according to the manufacturer's protocol. Additionally, the PBMCs, isolated as described above, were suspended in the RPMI-1640 medium (3 × 10^6^ PBMCs/mL) and incubated with the extracts (5-50 *μ*g/mL) for 24 h (in 96-well microplates, at 37°C, in a humidified atmosphere). Measurements were carried out analogously as described above.

### 2.7. Data Analysis

The results were reported as means ± SD (standard deviation) or ± SE (standard error) for the indicated number of experiments. The significance of differences between samples and controls was determined with one-way ANOVA (for chemical tests) or one-way ANOVA for repeated measures (for human plasma model), followed by post hoc Tukey's test for multiple comparisons. The correlations were evaluated using an *F*-test. All calculations were performed using the Statistica12Pl software for Windows (StatSoft Inc., Krakow, Poland) with *p* values less than 0.05 regarded as significant.

## 3. Results

### 3.1. Phytochemical Profiling

**Figure 1:**
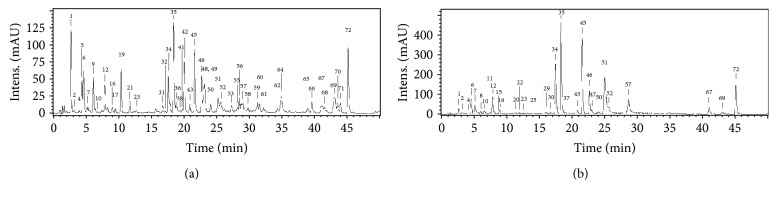
Representative UHPLC chromatograms at 280 nm of (a) DEF and (b) EAF extracts of *S. aucuparia* flowers. Peak numbers refer to those implemented in Table 1.

The LC-MS analysis revealed significant, extraction solvent-dependent differences in the chemical composition of the extracts (Figure 1, Table 1). The assay enabled the detection of 72 phenolic components (UHPLC peaks 1–72), the structures of 66 of which were fully or tentatively characterised based on the comparison of their chromatographic behaviour and ESI-MS^3^ fragmentation patterns (in a negative ionisation mode) with the literature data [11, 26–33] or reference standards, both commercial and isolated previously in our laboratory from various *Sorbus* species [34, 35]. The analytes represented a wide range of polyphenolic classes, including flavonols, pseudodepsides of quinic acid and shikimic acid, flavan-3-ol derivatives (catechins and proanthocyanidins), simple phenolic acids, phenylamides, flavanones, and flavalignans. The greatest chemical diversity was observed for MED, while its fractions (DEF, EAF, BF, and WR) obtained after sequential liquid-liquid partitioning were enriched in selected constituents, depending on the fractionation solvent. Among the analytes, only CHA (7) was found in all extracts. The fractionation has influenced also the quantitative profiles of the extracts (Table 2). The TPC levels determined by the Folin-Ciocalteu assay varied in a wide range of 111.7-597.6 mg GAE/g dw with the highest value observed for EAF. The primary constituents of the basic extract MED were mono- and dicaffeoylquinic acids with the dominating CHA and flavonol mono- and diglycosides with the prevailing IQ (35). In accordance with their polarity, flavonol monoglycosides were concentrated mainly in EAF and DEF, flavonol diglycosides in BF, and monocaffeoylquinic acids (CHA isomers) in BF and WR, while dicaffeoylquinic acids (cynarin isomers) and simple phenolic acids in DEF. The total contents of phenolics (TPH), calculated as a sum of individual analytes quantified by RP-HPLC-PDA, were in the range of 82.7-554.0 mg/g dw, with the highest concentration observed still for EAF. The TPH values were, however, significantly lower than the TPC levels. This difference might be related to the presence of proanthocyanidins—the compounds occurring in plants at various degrees of polymerisation and measureable by RP-HPLC only in the form of oligomers built from less than four flavan-3-ol monomers. Within this group, only low levels of flavan-3-ols (peaks: 6, 12, 32, 38) and B-type proanthocyanidin dimers (peaks: 2, 4, 10, and 37) were observed in the extracts. In consequence, the total contents of proanthocyanidins (TPA) determined by the *n*-butanol/HCl assay (11.4-380.2 mg CYE/g dw) might be considered as a measure of the content of higher flavan-3-ol oligomers.

### 3.2. Antioxidant Activity Assays against Multiple Oxidants

**Figure 2:**
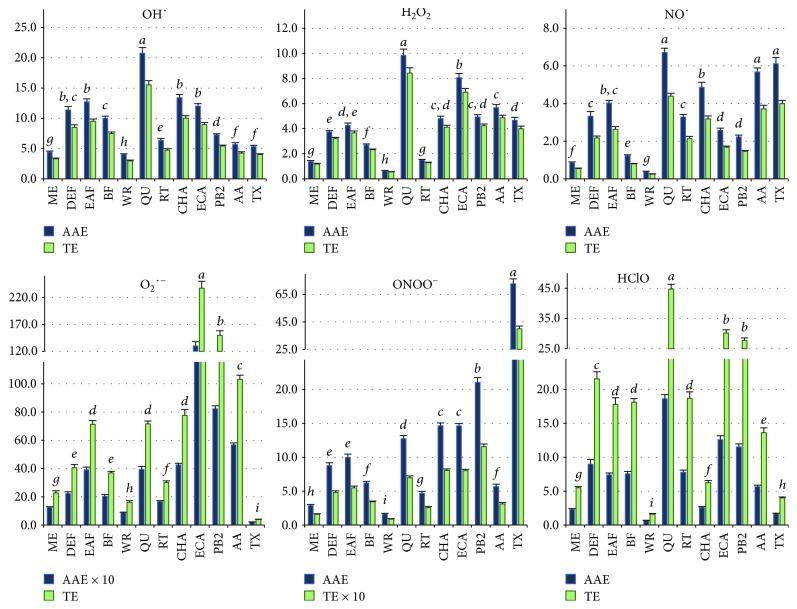
Scavenging activity of the *S. aucuparia* flower extracts, model polyphenols, and standard antioxidants on six different biologically relevant oxidants, expressed in equivalents of AA (mmol AAE/g dw) and TX (mmol TE/g dw). Values on particular charts labelled with the same italics (*a*-*i*) did not differ significantly at *α* = 0.05.

All of the investigated extracts showed significant and concentration-dependent ability to scavenge the six most common *in vivo*-relevant ROS (Figure 2). In terms of AA (primary antioxidant in human plasma) equivalents, the strongest effects were revealed towards OH^∙^, ONOO^–^, and HClO, while with the reference to TX (a synthetic analogue of vitamin E) towards OH^∙^, HClO, and O_2_^∙−^. In five of the tests (except the HClO-scavenging), the extract activity decreased in the same order, i.e., EAF>DEF>BF>MED>WR and significantly correlated (*p* < 0.05) with the amounts of polyphenols, i.e., TPC (*r* > 0.86) and/or TPH (*r* > 0.88) values (Table 3). In the O_2_^∙−^, H_2_O_2_, NO^∙^, and ONOO^–^-scavenging assays, the significant influence of total flavonoids (TFL; *r* > 0.88), total low-molecular-weight proanthocyanidins (TLPA; *r* > 0.83), and/or total caffeic acid derivatives (TCFA; *r* > 0.91) was also evidenced. In the case of HClO, the activity order was slightly different, i.e., DEF>BF≥EAF>MED>WR, but the correlation with the TPC levels was still significant (*r* = 0.96). As no correlation was found between the antioxidant activity parameters and TPA values, the extract effects might be attributed mainly to low-molecular-weight polyphenols, detectable by RP-HPLC. Indeed, the simultaneous analysis of model extract constituents, representing the main groups of *Sorbus* polyphenols (QU, RT, ECA, PB2, and CHA), revealed their high scavenging efficiency towards the analysed ROS, similar in terms of the order of magnitude to the most active extracts (Figure 2).

### 3.3. Protective Effects on Human Plasma Components

**Figure 3:**
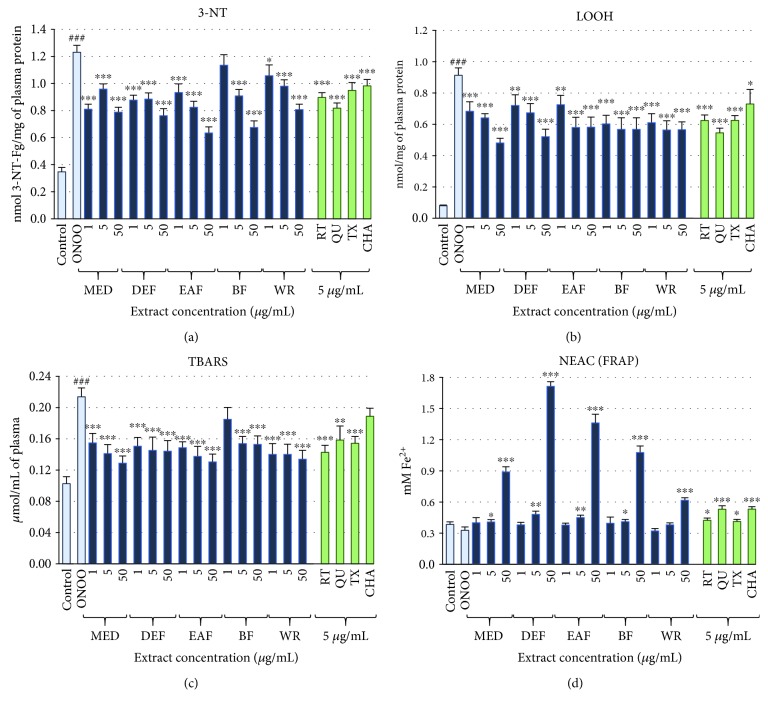
Effects of *S. aucuparia* flower extracts on human plasma exposed to oxidative stress: (a) effects on the nitration of plasma proteins and formation of 3-NT; (b, c) effects on the peroxidation of plasma lipids and formation of (b) LOOH and (c) TBARS; (d) effects on NEAC of plasma (measured by FRAP). Results presented as means ± SE (*n* = 12). Statistical differences: ^###^*p* < 0.001 for control plasma versus ONOO^–^-treated plasma (without the extracts); ^∗^*p* < 0.05, ^∗∗^*p* < 0.01, and ^∗∗∗^*p* < 0.001 for ONOO^–^-treated plasma in the presence of the extracts (1, 5, or 50 *μ*g/mL) or standards (5 *μ*g/mL) versus ONOO^–^-treated plasma in the absence of the extracts.

The addition of ONOO^–^ (100-150 *μ*M) to the plasma samples induced oxidative stress resulting in a considerable (*p* < 0.001) oxidative and nitrative damage to blood plasma components, confirmed by the measurements of specific biomarkers of protein nitration (3-NT, Figure 3(a)) and lipid peroxidation (LOOH and TBARS, Figures 3(b) and 3(c), respectively), as well as in a slight but statistically significant (*p* < 0.05) impairment of the NEAC of the plasma measured by the FRAP assay (Figure 3(d)). In comparison to the control plasma, in the ONOO^–^-treated samples, the NEAC was diminished by about 15%, and the oxidative damage was evidenced by an approximately 3.5-fold, 11-fold, and 2-fold increase in the levels of 3-NT, LOOH, and TBARS, respectively. In plasma samples incubated with ONOO^–^ in the presence of the analysed extracts (at 1-50 *μ*g/mL), the rate of oxidative and nitrative damage was significantly reduced (Figures 3(a)–3(c); *p* < 0.05). The antinitrative activity of the extracts was solvent-dependent, and the strongest effects were observed for MED, DEF, and EAF, with the highest inhibitory percentage (49%) found for EAF at 50 *μ*g/mL (Figure 3(a)). However, even at the lowest concentration (1 *μ*g/mL), these three extracts were able to diminish effectively (by about 24-33%) the ONOO^–^-induced protein nitration. Moreover, the antinitrative effects of the extracts were dose-dependent (except those of MED) and phenolic-dependent, which was evidenced by significant (*p* < 0.05) correlations between percentage inhibition of tyrosine nitration and phenolic contents (Table 4), especially the TCFA levels (*r* = 0.8220). All of the tested extracts protected also the plasma lipids against ONOO^–^-caused peroxidation (Figures 3(b) and 3(c); *p* < 0.05), regardless of the concentration levels. The strongest inhibitory effects—in the range of 21-35% at 1 *μ*g/mL—were revealed for the formation of LOOH that reflected the first stage of the peroxidation process. The influence of the extracts on the generation of TBARS, which measured the final stadium of the process, was slightly lower (11-32% of inhibition at 1 *μ*g/mL). The dose dependence was evident only for some of the extracts (e.g., for MED and DEF in the LOOH test), and the impact of phenolics was statistically proved (*p* > 0.05) only for the LOOH levels. In contrast, the ability of the extracts to normalise and/or enhance the NEAC status of plasma (Figure 3(d)) was strongly dose-dependent, and thus strong and statistically significant (*p* < 0.05) relationships were found between the percentage increase in the FRAP values of the oxidised plasma and the phenolic contents (Table 4), especially the TPC levels (*r* = 0.9864).

The observed effects of the extracts were in general comparable to (*p* > 0.05) or higher (*p* < 0.05) than those of positive phenolic standards—RT, CHA, and TX (Figures 3(a)–3(d)) applied at the same concentration. Even the activity of QU, one of the most powerful natural antioxidants, did not differ significantly (*p* > 0.05) in the majority of the tests from that observed for the most effective extracts (Figures 3(a)–3(c)). Only in the FRAP assay, two of the standards—QU and CHA—enhanced the NEAC of plasma more strongly than the extracts at the corresponding concentration (Figure 3(d); *p* < 0.05).

### 3.4. Inhibitory Effects on Proinflammatory Enzymes

The extracts inhibited the activity of LOX and HYAL in a concentration-dependent manner, but with different responses towards particular enzymes (Table 5). Considering the IC_50_ values expressed in *μ*g/U, most extracts and all standards were stronger inhibitors of LOX than of HYAL. The exceptions were BF and WR, showing similar inhibitory potential towards both enzymes. Different orders of potency were also observed in both tests, with BF being the most active towards HYAL and three extracts (DEF, EAF, and BF, not differing significantly in activity) the most effective towards LOX. Moreover, BF was a stronger HYAL inhibitor than all standards including IND, a potent nonsteroidal anti-inflammatory drug. In the LOX test, the activity of the most effective extracts was intermediate between that of IND and that of most of the phenolic standards except QU. The responses in this test were strongly TPC-dependent (*r* = –0.9733, *p* < 0.01) with some effects demonstrated for the main groups of phenolics, including TFL (*r* = −0.7379, *p* = 0.16), TCFA (*r* = –0.6844, *p* = 0.20), and TPH (*r* = –0.7694, *p* = 0.13). On the other hand, the activity of the extracts in the HYAL test was only slightly related to the TPA (*r* = –0.7927, *p* = 0.11) and not connected with the TPC levels (*r* = –0.0565, *p* = 0.93). Nevertheless, similar IC_50_ values obtained for the extracts and most of the model polyphenols (*p* < 0.05) might indicate strong synergic effects between the extract components. In contrast to their inhibitory potential towards LOX and HYAL, the extracts did not influence XO, the activity of which, in the presence and absence of the extracts in a wide range of concentrations, did not differ significantly (*p* > 0.05; results not shown).

### 3.5. Influence of the Extracts on Cell Viability

**Figure 4:**
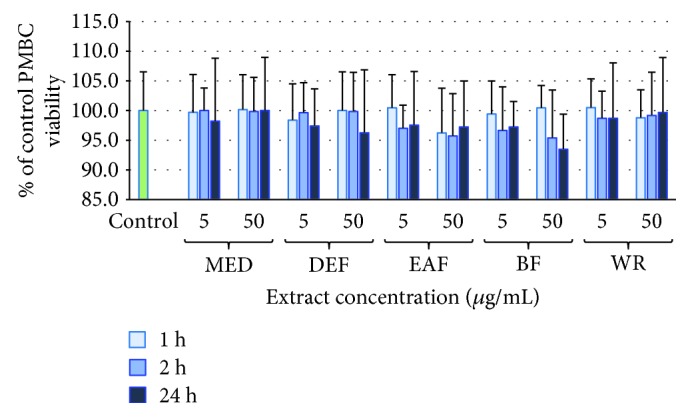
Viability of PBMCs after 1, 2, and 24 h of incubation with *S. aucuparia* flower extracts at 5 and 50 *μ*g/mL. All values are not statistically different (*p* > 0.05).

The potential cytotoxicity of the extracts was evaluated in a model of PBMCs after 1-24-hour incubation with the extracts at 5-50 *μ*g/mL. Regardless of the incubation time and extract concentration, the viability of the PBMCs treated with the extracts constituted 93.5-100.5% of that of the control (untreated) samples. Cellular safety of the extracts was evidenced by the lack of significant differences (*p* > 0.05) between the respective results (Figure 4).

## 4. Discussion

Standardised dry extracts from dietary and medicinal plants constitute the basis of modern phytotherapy as they contain concentrated active components and provide higher therapeutic effectiveness than unprocessed plant materials. Our previous studies had demonstrated that *S. aucuparia* flower is a promising candidate for cost-effective production of dry extracts rich in polyphenols [10, 14], but their phytochemical profiles and biological effects have remained inadequately recognised. In the present work, after fractionated extraction (which enabled enrichment of phenolic subfractions in active constituents) and subsequent LC-MS/MS profiling, we were able to detect and fully or tentatively identify 66 flower components, among which 52 and 50 structures were found for the first time in the analysed plant material and in the species *S. aucuparia*, respectively (Table 1). In contrast, only 17 constituents were observed earlier in the leaves [11] and 24 in the fruits of rowan [9]. A distinctive feature of the flower extracts was a vast diversity of the flavonol fraction (24 peaks) and high abundance of sexangularetin (8-methoxykaempferol) glycosides. As the derivatives of this flavonol are typical of bee pollen of rosaceous plants [36], they might be considered as a valuable marker for the identification of the origin of the extracts from the flowers. Flower-specific are also phenylamides (tricoumaroyl- and dicoumaroyl-caffeoyl spermidine isomers). This type of *N*-acylated biogenic amines, described here for the first time in the genus *Sorbus*, is usually reported in floral buds and reproductive organs with the suggested role in plant growth, flower development, and antimicrobial defence [33]. High analytical and chemotaxonomic value might be also ascribed to other new *Sorbus* constituents—flavalignans (three cinchonain I isomers). These compounds are phenylpropanoid-substituted flavanols in which two phenolic units are coupled by a two-bond lignin-like linkage [37]. Flavalignans are rare in nature, having been previously found, among others, in some species of *Acer*, *Cinchona*, and *Trichilia* [32, 37]. Although in the analysed *Sorbus* extracts flavalignans occurred at relatively low levels, the accumulated knowledge on their bioactivity suggests that by the additive and/or synergistic effects, they might influence the biological capacity of the extracts. According to Tang et al. [37], cinchonain I isomers are strong antioxidants, and their antiradical properties (towards DPPH) are up to four times stronger than those of (+)-catechin—one of the most effective antioxidants *in vitro* and *in vivo* [38]. Indeed, the cinchonains were concentrated in the most active fractions DEF and EAF (Figure 1, Table 2).

The quantitative survey of the *Sorbus* extracts revealed remarkable constancy of their polyphenolic profiles, reflected in similar levels of the main active components in flowers harvested in different years, i.e., 2009 [10] and 2015 (the present work). For example, the levels of TPC, TPA, TFA, and TCHA in the 2009 sample were 211.7, 111.6, 58.3, and 65.6 mg/g dw [10], while those reported here for the 2015 sample were 221.9, 110.9, 40.0, and 86.7 mg/g dw, respectively (Table 2). The intersample variations might be caused in part by different environmental (climatic) conditions in the year of harvest and in part by differences in the applied analytical protocols. For instance, the use of MS/MS detection in the present work enabled the identification of dicaffeoylquinic acid isomers, which then could have been accurately quantified using an authentic standard of cynarin (Figure 1, Table 2) instead of caffeic acid applied earlier [10]. Nevertheless, the relative stability of the chemical composition of the extracts indicates their great potential for industrial and phytotherapeutic applications.

The cooccurrence of high levels of flavonols and caffeic acid pseudodepsides in the investigated extracts suggests their possible significant biological effects in NCDs. Flavonols, especially QU glycosides, show a wide range of biological activities and are considered the most active compounds among flavonoids [39]. Their beneficial effects in NCDs depend largely on the nature of sugar units attached to the aglycones and on the other components of the plant matrix (emulsifiers) which may affect the solubility and bioavailability of the individual glycosides. One of the most effective flavonols is IQ, the bioavailability of which was up to 2.4-fold higher than that of QU and up to 6.4-fold higher than that of RT—the most abundant flavonoid in nature [40]. In consequence, ingestion of IQ as a pure compound (a 150 mg dose in humans) or in the plant matrix (a 62 mg/kg dose in rats) resulted in the plasma levels up to 5 *μ*M (1.5 *μ*g/mL) or 9.3 *μ*M (2.8 *μ*g/mL) of QU equivalents, respectively [40, 41]. Due to the advantageous bioavailability, IQ has been attracting increased attention, and its antioxidant, anti-inflammatory, anticarcinogenic, cardioprotective, antidiabetic, antiallergic, and neuropharmacological activity have been demonstrated ex vivo and *in vivo* [40]. Although IQ is widely distributed, its content in plant extracts is usually low; e.g., in the leaf extract of *Morus alba*, it amounts to 38.1 mg/g dw [40]. With the IQ levels up to 155.2 mg/g dw in EAF, the flower dry extracts of *S. aucuparia* appear thus promising sources of this valuable compound. Worth noting is also the abovementioned abundance of sexangularetin 3-glucoside in the analysed extracts (Table 2). Methoxylated flavonoids display improved intestinal absorption and enhanced resistance to the intestinal and hepatic metabolism and can be accumulated in mammalian tissues at up to 3.5-fold higher levels than their hydroxylated counterparts [42]. The relatively high bioavailability is also reported for caffeoylquinic acids, the dominant constituents of the *Sorbus* extracts. The plasma level of total monocaffeoylquinica acids and CHA after ingestion of green coffee bean extract may reach up to 14.8 *μ*M (5.2 *μ*g/mL) and 5.9 *μ*M (2.1 *μ*g/mL), respectively [43]. The best known source of these compounds is green coffee. The green coffee bean extract, standardised for total caffeoylquinic acids not less than 250 mg/g dw, is indicated mainly in the treatment of NCDs as antiatherogenic, antihypercholesteraemic, choleretic, antiobesity, hepatoprotective, and cardioprotective agent [44]. With the TCHA levels up to 169.0 mg/g dw (Table 2), the dry *Sorbus* extracts might be thus expected an attractive alternative for similar applications. The dominant groups of flavonols and caffeoylquinic acids were accompanied in the analysed extracts by flavanols and proanthocyanidins (Table 2), known antioxidant and anti-inflammatory plant constituents acting predominantly within the vascular system or the gastrointestinal tract, depending on the molecular mass and bioavailability [45]. The beneficial effects of proanthocyanidins in NCDs are attributed mainly to low-molecular-weight compounds and their ability to inhibit lipid peroxidation, reduce serum concentrations of proinflammatory cytokines and related inflammation of blood vessel walls, diminish capillary fragility and permeability, and decrease blood pressure [45]. As this group occurs in the analysed *Sorbus* extracts at relatively low levels, its possible influence on the extract activity *in vivo* is expected to be inferior to that of the dominant flavonols and caffeoylquinic acids. Nevertheless, considering possible additive and/or synergistic effects, the composition of the leaf extracts appears to be promising in the context of their potential application as antioxidant and anti-inflammatory agents, especially in the prevention of NCDs.

In our previous works, we had reported strong antiradical activity of the flower extracts of *S. aucuparia* towards synthetic free radicals: DPPH and ABTS, as well as their FRAP reactivity and ability to inhibit linoleic acid peroxidation in nonbiological systems [10, 14]. The present study investigated the interactions of the extracts with six radical and nonradical ROS involved in generation of oxidative stress *in vivo*. The primary ROS expressed by the human cells, e.g., by stimulated neutrophils during the inflammatory process, is O_2_^∙–^ [3]. The ROS-mediated oxidative damage to biomolecules *in vivo* may result from the overproduction of O_2_^∙–^ itself or from the oxidants derived in the downstream reaction cascade of O_2_^∙–^, such as H_2_O_2_, HClO, OH^∙^, and ONOO^–^. The most stable and diffusive form of ROS is H_2_O_2_, the harmful effects of which are connected with its selective reactivity with cysteine residues in proteins and ability to generate OH^∙^, the most reactive species in chemistry, able to attack and damage almost every molecule in the living cell [3, 46]. Highly destructive and nonselective oxidants are also HClO, the key antimicrobial agent involved, however, in the pathogenesis of many diseases, and ONOO^–^. The latter one, formed in the reaction between O_2_^∙–^ and NO^∙^, causes protein nitration—one of the most dangerous processes in the cells [47]. The case of NO^∙^ is a good example of a dual nature of ROS. When produced by endothelial NO synthase (eNOS) in the vessel endothelium, it plays an important role in vessel dilatation and inflammatory protection (inhibition of leukocyte adhesion), but if synthesised in stimulated macrophages by inducible NO synthase (iNOS), it makes contact with O_2_^∙–^ and rapidly forms the toxic ONOO^–^ [48]. We found that the *Sorbus* extracts were able to inactivate all of the abovementioned ROS of physiological importance, and the effectiveness of the most active analytes was comparable to that of AA—the primary antioxidant of human plasma (Table 3). The correlation studies and experiments with the model compounds proved that the observed effects are determined by polyphenols (Table 4). The polyphenol-rich *Sorbus* extracts might be therefore expected to reduce the consequences of oxidative stress in biological systems.

This hypothesis was verified in an *in vitro* model of human plasma exposed to oxidative stress. The stress conditions were induced by ONOO^–^, one of the strongest oxidative and nitrative agents operating *in vivo*, involved in the pathophysiology of various inflammatory, neurodegenerative, metabolic, and especially cardiovascular disorders [49]. Although it is a short-lived oxidant, it easily crosses biological membranes and generates or decomposes into highly reactive ROS, such as OH^∙^, CO_3_^∙–^, NO^∙^, or ^∙^NO_2_, forming an aggressive cocktail of oxidants able to interact with the most critical biomolecules including plasma proteins and lipids [47]. The destructive effect of ONOO^–^ is reflected, among others, in the increased plasma levels of nitrative/oxidative stress biomarkers such as 3-NT, LOOH, or TBARS and in the decrease of the NEAC of plasma [50]. The levels of these parameters have been connected with the progress or worse prognosis of many NCDs, including cardiovascular events, hypertension, diabetes, rheumatoid arthritis, chronic hepatitis, multiple sclerosis, and Alzheimer's disease [51].

In our experimental model, the plasma samples were treated with 100-150 *μ*M ONOO^–^ (depending on the test), which was sufficient to induce measurable changes in the levels of the oxidative stress biomarkers. Moreover, the applied concentrations correspond to the levels of ONOO^–^ that can be reached *in vivo* in local compartments, e.g., during a serious inflammation of blood vessels [47]. The results indicated that the extracts might indeed have a beneficial impact on the plasma antioxidant status and protect its components against the harmful effect of oxidative stress (Figure 2). The significant effects obtained for the model extract constituents and the results of correlation studies (Table 5) suggest that low-molecular-weight polyphenols play a crucial role in the observed activity. On the other hand, worse correlation parameters and weaker dose dependency than observed in nonbiological models might indicate the impact of some other factors, such as the interactions between endogenous plasma constituents and the *Sorbus* phenolics. For example, the binding of polyphenols to proteins may suppress their antioxidant properties, and these masking effects may depend both on proteins and on polyphenols [15]. It is symptomatic that the relatively high correlation was observed by us in the 3-NT and FRAP tests. We suppose that the formation of the polyphenolic adducts may protect proteins against nitration on a mechanistic way, while the suppression of the direct reactivity of the adducts with oxidants may be less pronounced in the case of small molecules such as ferric ions (FRAP assay) than lipid-derived radicals formed in chain reactions during lipid peroxidation (LOOH and TBARS tests).

Nevertheless, the mechanism behind the observed protective effects was probably a direct and polyphenol-related scavenging of various ROS operating in plasma under the applied experimental conditions. It is consistent with the noticeable scavenging potential of the extracts towards ONOO^–^ and some of the ONOO^–^-derived secondary radicals (OH^∙^, NO^∙^), as well as with the accumulating evidence of antiradical effects of polyphenols against CO_3_^∙–^ and ^∙^NO_2_, formed in the fundamental reaction of ONOO^–^ (with CO_2_) in biological systems [52]. The protective effects of the extracts in plasma were statistically significant (*p* < 0.05) at the concentrations as low as 1-5 *μ*g/mL, equivalent to 0.11-3.0 *μ*g GAE/mL, depending on the TPC value. Taking into account the results of the bioavailability studies that reported plasma levels of the model polyphenols (IQ, CHA, and ECA) in the range of 5-15 *μ*M (1.5-5.2 *μ*g/mL) [40, 41, 43], these concentrations seem possible to be achieved *in vivo* after ingestion of the analysed extracts. At these concentrations, their polyphenolic constituents might support the endogenous nonenzymatic antioxidant system by, among others, additive or sparing effects on the primary plasma antioxidant—ascorbic acid. It is well established that in various pathologic conditions, the plasma ascorbate level is below the optimal value of 50 *μ*M (8.8 *μ*g/mL) adequate to retain the redox homeostasis, and this decrease is one of the preconditions for the development of oxidative stress-related NCDs [53]. For instance, Deicher et al. [54] indicated that the ascorbate levels less than 32 *μ*M (5.6 *μ*g/mL) are associated with the increased risk of adverse cardiovascular events including myocardial infarction and death. Considering the protective effects of the *Sorbus* extracts in plasma at physiological levels as well as their antioxidant capacity towards the *in vivo*-relevant oxidants comparable to that of AA, the extracts might be expected to reduce the negative consequences of the disturbed redox homeostasis *in vivo* at the appropriate oral doses. In the context of future *in vivo* applications, it is also of note that at the wide range of concentrations (1-50 *μ*g/mL), the extracts did not exhibit any prooxidant effects, did not deteriorate the viability of PBMCs, and thus may be regarded as safe (Figure 4).

Oxidative stress *in vivo* is closely linked with inflammation, and simultaneous influence on both processes is crucial for the effectiveness of antioxidant therapies [3]. In the present study, we investigated the anti-inflammatory potential of *Sorbus* extracts by determining their inhibitory activity towards three model enzymes involved in inflammation, which are some of the targets proposed for the treatment of inflammatory-related complaints [55, 56]. The first enzyme—LOX—is a prooxidant agent belonging to the family of lipoxygenases, enzymes catalysing the incorporation of dioxygen molecules into polyunsaturated fatty acids and formation of key chemokines and ROS, such as leukotrienes and O_2_^∙–^, associated with the development of numerous NCDs, e.g., atherosclerosis, myocardial infarction/reperfusion injury, rheumatoid arthritis, and cancer [56]. The second enzyme, HYAL, known as a spreading factor, hydrolyses hyaluronic acid, an important constituent of, i.e., endothelial surface layer, the disruption of which causes endothelium dysfunction and increases the instability of the atherosclerotic plate [55]. Our study also included XO, the prooxidant, O_2_^∙–^-generating enzyme that plays an important role in various ischemic and inflammatory diseases [57]. The results indicated that the extracts are inactive towards XO but are potent inhibitors of LOX and HYAL (Table 5). The inhibitory potential of the most active extracts was at most 1.5-fold lower than that of indomethacin—a commercial nonsteroidal anti-inflammatory drug. The activity towards LOX was found to be dependent on low-molecular-weight polyphenols (relatively well bioavailable), which is promising in the context of possible future application of the extracts. The anti-HYAL capacity was in turn related to poorly bioavailable condensed proanthocyanidins (TPA); the systemic effects of the TPA-rich extracts, especially BF, are thus less likely.

## 5. Conclusion

The present paper is the first detailed study of flower extracts of *S. aucuparia* and provides new insights into their phytochemical composition, standardisation, biological activity, and cellular safety. The results revealed that the extracts accumulate a vast diversity of bioactive polyphenols with the levels and structures promising for the use in the prophylaxis or adjunctive therapy of oxidative stress- and inflammation-related diseases. Some of the phenolic components, such as sexangularetin glycosides, phenylamides, and flavalignans, might be of chemotaxonomic importance and serve as analytical markers for the authentication of the extract origin. The main constituents, such as flavonols and phenolic pseudodepsides of quinic and shikimic acids, which were found responsible for antioxidant and anti-inflammatory activity of the extracts, might be in turn recommended as standardisation targets for routine quality control. Among the extracts, the defatted methanol-water (7 : 3, *v/v*) extract and its diethyl ether and ethyl acetate fractions appear to be the most advantageous for biological applications, considering both the yield and the activity in comparison to AA and IND. Some of their expected health benefits might be associated with the ability to neutralise multiple oxidants operating in human plasma, protect the plasma components (both proteins and lipids) against oxidative and nitrative damage, and increase the NEAC of plasma, as well as inhibit proinflammatory enzymes, especially LOX. However, although significant antioxidant protection was showed at physiological levels, the real extract effects should be verified *in vivo* in animal and clinical studies. As the observed effects might be related to possible antiatherogenic, anticoagulant, and antiplatelet functions of the extracts or their ability to influence endothelium, these issues should be first addressed in future research.

## Figures and Tables

**Table 1:** UHPLC-PDA-ESI-MS^3^ identification data of polyphenols detected in the dry extracts from *S. aucuparia* flowers.

No.	Analyte	*R*_t_ (min)	UV *λ*_max_ (nm)	[M–H]^–^ (m/z)	MS^2^/MS^3^ fragmentation	Extract	References
1	Protocatechuic acid*^a^*	3.0	259, 294	153		DEF, EAF	
2	(Epi)catechin-B-(epi)catechin^*b*,*c*^	3.4	280	577	451 (46), 425 (100), 407 (33)	DEF, EAF	[29]
3	3-*O*-Caffeoylquinic acid (neochlorogenic acid)*^a^*	3.5	216, 324	353	191 (100), 179 (30), 135 (3)	ME, BF, WR	[9, 11, 26]
4	(Epi)catechin-B-(epi)catechin^*b*,*c*^	4.2	279	577	451 (60), 425 (100), 407 (28)	ME, DEF, EAF	[29]
5	*p*-Hydroxybenzoic acid*^a^*	4.6	255	137		DEF	
6	(+)-Catechin^*a*,*b*^	4.9	278	289	245 (100), 205 (21)	ME, DEF, EAF	
7	5-*O*-Caffeoylquinic acid (chlorogenic acid, CHA)*^a^*	5.4	216, 325	353	191 (100), 179 (3)	ME, DEF, EAF, BF, WR	[9, 11, 26]
8	4-*O*-Caffeoylquinic acid (cryptochlorogenic acid)*^a^*	6.3	216, 325	353	191 (50), 179 (49), 173 (100)	ME, EAF, BF, WR	[26]
9	Caffeic acid*^a^*	6.4	215, 323	179		DEF	
10	Procyanidin B2 (PB2)*^b^*	6.8	280	577	451 (44), 425 (100), 407 (23)	DEF, EAF, BF	[29]
11	Unidentified	7.8	261	360	313 (100), 151 (49)	EAF, BF	
12	(–)-Epicatechin (ECA)^*a*,*b*,*c*^	8.1	279	289	245 (100), 205 (32)	ME, DEF, EAF	
13	Eriodictyol *O*-hexoside^*b*,*c*^	8.2	279, 330sh	449	287 (100), 269 (25), 259 (31)	BF	[31]
14	Unidentified	8.4	249, 297	641	461 (17), 317 (100)	ME, BF	
15	5*-O-p*-Coumaroylquinic acid^*b*,*c*^	9.0	207, 311	337	191 (100), 163 (13)	EAF, BF	[26]
16	5-*O*-Caffeoylshikimic acid^*b*,*c*^	9.2	211, 325	335	291 (25), 179 (100), 135 (11)	ME, DEF, EAF, BF	[30]
17	Vanillic acid^*a*,*b*,*c*^	9.6	260, 293	167		DEF	
18	Sexangularetin di-*O*-hexoside^*b*,*c*^	9.7	272, 326	639	519 (25), 477 (100), 315 (21)	ME, BF	[31]
19	*p*-Coumaric acid*^a^*	10.5	222, 309	163		DEF	
20	Quercetin 3-*O*-*β*-sophoroside*^a^*	11.6	255, 353	625	463 (23), 445 (39), 301 (100)	ME, EAF, BF	
21	Coumaric acid isomer^*b*,*c*^	11.9	293	163		DEF	
22	Quercetin *O*-dihexoside^*b*,*c*^	12.3	255, 352	625	463 (35), 445 (60), 301 (100)	ME, EAF, BF	[31]
23	4-*O*-Feruloylquinic acid^*b*,*c*^	12.7	214, 323	367	191 (37), 179 (100), 135 (35)	DEF, EAF	[26]
24	Sexangularetin *O*-dihexoside^*b*,*c*^	14.2	270, 337	639	477 (16), 459 (86), 315 (100)	BF	[31]
25	Quercetin *O*-pentosylhexoside^*b*,*c*^	14.3	270, 340	595	463 (12), 445 (25), 301 (100)	EAF, BF	[31]
26	Kaempferol *O*-dihexoside^*b*,*c*^	15.0	267, 340	609	447 (6), 429 (27), 285 (100)	BF	[31]
27	Kaempferol *O*-dihexoside^*b*,*c*^	15.5	266, 343	609	447 (13), 429 (86), 285 (100)	BF	[31]
28	Quercetin *O*-hexosylpentoside^*b*,*c*^	16.2	269, 342	595	433 (10), 415 (35), 301 (100)	ME, BF	[31]
29	Sexangularetin *O*-rhamosylhexoside^*b*,*c*^	16.3	273, 333	623	477 (15), 459 (61), 315 (100)	ME, EAF, BF	[31]
30	Quercetin *O*-rhamosylhexoside^*b*,*c*^	16.9	255, 353	609	301 (100)	ME, EAF, BF	[31]
31	Cinchonain I isomer^*b*,*c*^	17.0	280	451	341 (100)	DEF	[32]
32	(Epi)catechin derivative^*b*,*c*^	17.3	279	483	451 (45), 341 (17), 289 (100)	DEF	
33	Quercetin 3-*O*-(6^″^-*O*-*α*-l-rhamnosyl)-*β*-d-glucoside (rutin, RT)*^a^*	17.5	256, 353	609	301 (100)	ME, BF	[11]
34	Quercetin 3-*O*-*β*-d-galactoside (hyperoside)*^a^*	17.6	255, 354	463	301 (100)	ME, DEF, EAF, BF	[11]
35	Quercetin 3-*O*-*β*-d-glucoside (isoquercitrin, IQ)*^a^*	18.6	256, 353	463	301 (100)	ME, DEF, EAF, BF	[11]
36	Unidentified	19.0	312	193		DEF	
37	(Epi)catechin-B-(epi)catechin^*b*,*c*^	19.2	279	577	451 (60), 425 (100), 407 (28)	EAF	[29]
38	(Epi)catechin derivative^*b*,*c*^	19.4	256	483	451 (77), 341 (37), 289 (100)	DEF	
39	Unidentified	19.6	280, 333sh	597	477 (75), 387 (75), 357 (100)	BF	
40	Kaempferol *O*-rhamosylhexoside^*b*,*c*^	19.8	277, 335	593	447 (11), 285 (100)	BF	[31]
41	Unidentified^*b*,*c*^	19.9	282	519	309 (100)	DEF	
42	Ferulic acid^*a*,*b*,*c*^	20.2	217, 321	193		DEF	
43	Kaempferol *O*-hexoside^*b*,*c*^	21.0	264, 344	447	285 (100)	DEF, EAF	[31]
44	Eriodictyol *O*-glucuronide^*b*,*c*^	21.5	283	463	287 (100)	ME, BF	[9]
45	Sexangularetin 3-*O*-*β*-d-glucoside (sorbaroside)*^a^*	21.8	270, 350	477	315 (100)	ME, DEF, EAF, BF	
46	Kaempferol 3-*O*-*β*-d-glucoside (astragalin)^*a*,*b*,*c*^	22.9	265, 343	447	285 (100)	ME, DEF, EAF, BF	
47	Unidentified	23.2	273, 350	507	491 (100), 345 (40), 329 (36)	EAF, BF	
48	Cinchonain I isomer^*b*,*c*^	23.2	279	451	341 (100), 299 (23)	ME, DEF	[32]
49	Cinchonain I isomer^*b*,*c*^	23.3	279	451	341 (100)	ME, DEF	[32]
50	Quercetin acetylhexoside^*b*,*c*^	24.3	255, 350	505	463 (21), 301 (100)	ME, DEF, EAF, BF	[28]
51	3,5-*O*-Dicaffeoylquinic acid^*b*,*c*^	25.4	217, 326	515	353 (100), 191 (100)*^d^*, 179 (47)*^d^*	ME, DEF, EAF, BF	[27]
52	Caffeic acid derivative^*b*,*c*^	25.9	218, 328	437	377 (30), 275 (100), 179 (5)	ME, DEF, EAF	
53	Cinchonain I isomer^*b*,*c*^	27.4	281	451	341 (100)	DEF	[32]
54	Sexangularetin *O*-acetylhexoside^*b*,*c*^	27.9	271, 348	519	315 (100), 301 (60)	ME, BF	[28]
55	Quercetin *O*-acetylhexoside^*b*,*c*^	28.3	260, 348	505	463 (50), 301 (100)	DEF	[28]
56	Sexangularetin *O*-acetylhexoside^*b*,*c*^	28.5	271, 333	519	505 (32), 315 (100)	ME, DEF	[28]
57	4,5-*O*-Dicaffeoylquinic acid^*b*,*c*^	28.9	218, 325	515	353 (100), 179 (60)*^d^*, 173 (100)*^d^*	ME, DEF, EAF, BF	[27]
58	Kaempferol *O*-acetylhexoside^*b*,*c*^	30.0	265, 335	489	327 (12), 285 (100)	DEF	[28]
59	Cinchonain I isomer^*b*,*c*^	31.4	280	451	341 (100)	DEF	[32]
60	3-*O*-Caffeoyl-5-*O*-feruloylquinic acid^*b*,*c*^	31.7	308	529	367 (100), 191 (100)*^d^*, 179 (70)*^d^*	DEF	[27, 30]
61	3-*O*-Feruloyl-5-*O*-caffeoylquinic acid^*b*,*c*^	32.8	322	529	367 (100), 353 (84), 193 (100)*^d^*	DEF	[27, 30]
62	3-*O*-Caffeoyl-4-*O*-*p*-coumaroilquinic acid^*b*,*c*^	34.4	324	499	337 (100), 179 (100)*^d^*, 173 (97)*^d^*	DEF	[27, 30]
63	Caffeic acid derivative^*b*,*c*^	34.9	218, 328	437	377 (30), 275 (100), 179 (5)	BF	
64	Quercetin*^a^*	35.0	268, 364	301		DEF	
65	Tricoumaroyl spermidine isomer^*b*,*c*^	39.0	285	582	462 (100), 342 (12)	DEF	[33]
66	Dicoumaroyl-caffeoyl spermidine isomer^*b*,*c*^	39.7	288	598	478 (70), 462 (100), 342 (26)	DEF	[33]
67	Dicoumaroyl-caffeoyl spermidine isomer^*b*,*c*^	41.1	313	598	478 (47), 462 (100), 342 (30)	ME, DEF, EAF	[33]
68	Tricoumaroyl spermidine isomer^*b*,*c*^	41.6	293	582	462 (100), 342 (14)	DEF	[33]
69	Tricoumaroyl spermidine isomer^*b*,*c*^	43.0	289	582	462 (100), 342 (9)	DEF, EAF	
70	Kaempferol*^a^*	43.6	272, 373	285		DEF	
71	Tricoumaroyl spermidine isomer^*b*,*c*^	44.1	291	582	462 (100), 342 (12)	DEF	[33]
72	Tricoumaroyl spermidine isomer^*b*,*c*^	45.2	293	582	462 (100), 342 (24)	ME, DEF, EAF	[33]

*R*_t_: retention time. UV *λ*_max_: absorbance maxima in PDA spectra. [M–H]^–^: pseudomolecular ions in MS spectra recorded in a negative mode. MS^2^: secondary ions (the most abundant ions were subjected to MS^3^ fragmentation). Intensities of particular ions are given in parentheses. The nomenclature of the pseudodepsides of quinic acid and shikimic acid is given according to IUPAC [26, 27, 30]. *^a^*Compounds identified with authentic standards. *^b^*Compounds detected for the first time in *S. aucuparia* flowers. *^c^*Compounds detected for the first time in *S. aucuparia*. *^d^*MS^3^ ions.

**Table 2:** Quantitative profile of the *S. aucuparia* flower dry extracts (mg/g dw).

	MED	DEF	EAF	BF	WR
TPC (GAE)	221.9 ± 6.7*^d^*	533.3 ± 3.7*^b^*	597.6 ± 4.6*^a^*	485.0 ± 12.7*^c^*	111.7 ± 4.5*^e^*
TPH	137.5 ± 2.3*^d^*	300.6 ± 6.8*^b^*	559.6 ± 8.5*^a^*	248.7 ± 6.7*^c^*	82.7 ± 1.3*^e^*
TFL	40.0 ± 1.4*^d^*	130.7 ± 1.2*^b^*	333.9 ± 4.4*^a^*	115.2 ± 1.8*^c^*	2.9 ± 0.2*^e^*
SQ	4.4 ± 0.1*^b^*	n.d.	1.1 ± 0.1*^c^*	21.6 ± 1.2*^a^*	0.66 ± 0.04*^d^*
HY	6.2 ± 0.2*^d^*	24.2 ± 1.1*^b^*	79.1 ± 3.7*^a^*	12.2 ± 0.5*^c^*	0.11 ± 0.01*^e^*
IQ	11.7 ± 0.5*^d^*	56.2 ± 1.8*^b^*	155.2 ± 4.4*^a^*	21.6 ± 1.7*^c^*	0.07 ± 0.01*^e^*
GS	4.6 ± 0.2*^d^*	18.1 ± 0.7*^b^*	57.6 ± 2.1*^a^*	7.5 ± 0.3*^c^*	n.d.
RT	3.5 ± 0.1*^b^*	n.d.	3.4 ± 0.1*^b^*	18.1 ± 0.3*^a^*	0.34 ± 0.01*^c^*
TCFA (TCHA+CFA)	88.7 ± 3.3*^c^*	91.2 ± 4.1*^c^*	181.9 ± 5.5*^a^*	126.3 ± 4.4*^b^*	77.4 ± 3.1*^d^*
TCHA	86.7 ± 4.8*^c^*	57.5 ± 3.3*^e^*	169.0 ± 3.6*^a^*	108.3 ± 4.2*^b^*	76.3 ± 2.9*^d^*
NCHA	14.6 ± 0.5*^b^*	1.4 ± 0.1*^d^*	2.3 ± 0.1*^c^*	15.1 ± 0.6^*a*,*b*^	16.5 ± 0.8*^a^*
CHA	49.7 ± 1.4*^b^*	5.5 ± 0.2*^d^*	19.3 ± 0.8*^c^*	79.1 ± 2.8*^a^*	47.7 ± 1.9*^b^*
CCHA	8.6 ± 0.7*^b^*	1.2 ± 0.1*^d^*	2.9 ± 0.1*^c^*	13.9 ± 0.8*^a^*	10.2 ± 0.6*^b^*
1-CHA	2.2 ± 0.1*^b^*	n.d.	n.d.	4.9 ± 0.2*^a^*	2.0 ± 0.2*^b^*
CNE	11.6 ± 0.6*^c^*	49.3 ± 2.7*^b^*	144.5 ± 6.9*^a^*	n.d.	n.d.
CFA	1.9 ± 0.1*^d^*	33.7 ± 1.4*^a^*	12.9 ± 0.5*^c^*	18.1 ± 0.4*^b^*	1.0 ± 0.1*^e^*
HCA	3.5 ± 0.2*^c^*	8.9 ± 0.5*^a^*	6.4 ± 0.2*^b^*	4.3 ± 0.2*^c^*	1.2 ± 0.1*^d^*
HBA	1.1 ± 0.1^*c*,*d*^	38.2 ± 1.3*^a^*	2.2 ± 0.1^*b*,*c*^	0.62 ± 0.04*^d^*	0.71 ± 0.06*^d^*
TPA (CYE)	110.9 ± 2.2^*b*,*c*^	11.4 ± 0.3*^e^*	103.0 ± 2.8*^c^*	382.0 ± 4.3*^a^*	52.8 ± 1.1*^d^*
TLPA	2.1 ± 0.1*^c^*	9.1 ± 0.4*^b^*	22.5 ± 1.3*^a^*	2.3 ± 0.1*^c^*	0.59 ± 0.04*^d^*
LG	n.d.	2.7 ± 0.1*^a^*	0.76 ± 0.05*^b^*	n.d.	n.d.
SP	2.3 ± 0.2*^c^*	19.9 ± 1.2*^a^*	5.6 ± 0.2*^b^*	n.d.	n.d.

Results are presented as means ± SD (*n* = 3). For each parameter, different superscript letters indicate significant differences (*p* < 0.05). Additional abbreviations: n.d.: not detected; SQ: quercetin 3-*O*-sophoroside; HY: hyperoside; GS: sexangularetin 3-*O*-glucoside; NCHA: neochlorogenic acid (3-*O*-caffeoylquinic acid); CCHA: cryptochlorogenic acid (4-*O*-caffeoylquinic acid); 1-CHA: 1-*O*-caffeoylquinic acid; CNE: total content of dicaffeoylquinic acids (cynarin isomers); CFA: total content of caffeic acid derivatives other than TCHA; HCA: total content of simple hydroxycinnamic acids; HBA: total content of simple hydroxybenzoic acids; LG: total content of flavalignans; SP: total content of phenolic amides (spermidine derivatives). The highest levels for each parameter are printed in bold.

**Table 3:** Correlation (*r*) coefficients and probability (*p*) values of linear relationships between phenolic contents of the extracts and their activity parameters towards multiple oxidants.

*r* (*p*) for	TPC	TPH	TFL	TCFA	TCHA	TPA	TLPA
AAE (O_2_^∙^)	**0.8784 (0.050)** ^∗^	**0.9992 (0.000)** ^∗∗∗^	**0.9976 (0.000)** ^∗∗∗^	**0.9106 (0.032)** ^∗^	0.4419 (0.456)	0.0423 (0.946)	**0.9534 (0.012)** ^∗^
AAE (OH^∙^)	**0.9885 (0.001)** ^∗∗∗^	**0.8864 (0.045)** ^∗^	0.8569 (0.064)	0.7528 (0.142)	0.4567 (0.439)	0.1515 (0.808)	0.7677 (0.130)
AAE (H_2_O_2_)	**0.9796 (0.003)** ^∗∗^	**0.9161 (0.029)** ^∗^	**0.8847 (0.046)** ^∗^	0.7326 (0.159)	0.4455 (0.452)	0.0192 (0.976)	**0.8314 (0.081)** ^∗^
AAE (NO^∙^)	0.8666 (0.057)	**0.9176 (0.028)** ^∗^	**0.8863 (0.045)** ^∗^	0.6486 (0.236)	0.4001 (0.504)	0.2778 (0.651)	**0.9183 (0.028)** ^∗^
AAE (ONOO^–^)	**0.9773 (0.004)** ^∗∗^	**0.9170 (0.028)** ^∗^	**0.8853 (0.046)** ^∗^	0.7308 (0.161)	0.4419 (0.456)	0.0105 (0.987)	0.8343 (0.079)
AAE (HClO)	**0.9627 (0.009)** ^∗∗^	0.7162 (0.174)	0.6722 (0.214)	0.5734 (0.312)	0.2302 (0.709)	0.2290 (0.711)	0.5573 (0.329)

Activity and concentration parameters according to Figure 2 and Tables 2 and 3. Asterisks mean statistical significance of the estimated linear relationships (^∗^*p* < 0.05, ^∗∗^*p* < 0.01, and ^∗∗∗^*p* < 0.001). All statistically significant relationships are printed in bold. The (*r*) and (*p*) values for TE towards individual oxidants were the same as given for AAE.

**Table 4:** Correlation (*r*) coefficients and probability (*p*) values of linear relationships between phenolic contents of the extracts and their antioxidant activity parameters in human plasma (*n* = 15).

*r* (*p*) for	TPC	TPH	TFL	TCFA	TCHA	TPA	TLPA
3-NT	**0.7833 (0.001)** ^∗∗∗^	**0.7729 (0.001)** ^∗∗∗^	**0.7153 (0.003)** ^∗∗^	**0.8220 (0.000)** ^∗∗∗^	**0.8068 (0.000)** ^∗∗∗^	**0.5766 (0.024)** ^∗^	**0.6393 (0.010)** ^∗∗^
LOOH	**0.5439 (0.036)** ^∗^	0.4574 (0.086)	0.3429 (0.211)	**0.5697 (0.027)** ^∗^	**0.5477 (0.035)** ^∗^	0.3945 (0.146)	0.2948 (0.286)
TBARS	0.2978 (0.281)	0.3596 (0.188)	0.3072 (0.265)	0.4111 (0.128)	0.4323 (0.108)	0.0375 (0.895)	0.3525 (0.198)
FRAP	**0.9864 (0.000)** ^∗∗∗^	**0.9367 (0.007)** ^∗∗∗^	**0.8572 (0.000)** ^∗∗∗^	**0.9295 (0.000)** ^∗∗∗^	**0.8717 (0.000)** ^∗∗∗^	**0.5394 (0.038)** ^∗^	**0.7963 (0.000)** ^∗∗∗^

Activity and concentration parameters according to Figure 3 and Table 2. Asterisks mean statistical significance of the estimated linear relationships (^∗^*p* < 0.05, ^∗∗^*p* < 0.01, and ^∗∗∗^*p* < 0.001). All statistically significant relationships are printed in bold.

**Table 5:** Inhibition of the proinflammatory enzymes.

Analyte	LOX		HYAL	
	IC_50_ (*μ*g/mL)^∗^	IC_50_ (*μ*g/U)^∗∗^	IC_50_ (*μ*g/mL)^∗^	IC_50_ (*μ*g/U)^∗∗^
MED	188.1 ± 6.8*^e^*	6.94	18.3 ± 0.7*^f^*	16.0
DEF	91.6 ± 3.6*^b^*	3.38	25.3 ± 0.9*^h^*	22.2
EAF	89.8 ± 4.3*^b^*	3.31	12.4 ± 0.5*^c^*	10.9
BF	96.3 ± 3.7^*b*,*c*^	3.55	4.1 ± 0.2*^a^*	3.62
WR	265.3 ± 7.2*^f^*	9.79	11.3 ± 0.5*^c^*	9.94
QU	58.3 ± 2.3*^a^*	2.15	15.6 ± 0.9^*d*,*e*^	13.7
RT	104.8 ± 4.1*^c^*	3.86	23.2 ± 1.6*^g^*	20.4
CHA	114.3 ± 5.2*^d^*	4.21	16.5 ± 0.7*^e^*	14.5
ECA	90.6 ± 3.1*^b^*	3.34	14.3 ± 0.7*^d^*	12.5
PB2	77.0 ± 1.8*^b^*	2.84	12.8 ± 0.6*^d^*	11.2
IND	63.0 ± 2.7*^a^*	2.32	8.5 ± 0.4*^b^*	7.46

Results are presented as means ± SD (*n* = 3) calculated per dry weight of the extract or standard. For extract codes, see Table 1. Different superscripts in each column indicate significant differences in the means at *p* < 0.05. ^∗,∗∗^Inhibition concentration (amount of analyte needed for 50% inhibition of enzyme activity) expressed as follows: ^∗^in *μ*g of the dry extract or standard/mL of the enzyme solution; ^∗∗^in *μ*g of the extracts/enzyme unit (U).

## Data Availability

The source data used to support the findings of this study are available from the corresponding author upon request.

## Abbreviations

dw: Dry weight
MED: Defatted methanol-water (7 : 3, *v/v*) extract
DEF: Diethyl ether fraction
EAF: Ethyl acetate fraction
BF: *n*-Butanol fraction
WR: Water residue
NCDs: Noncommunicable (chronic) diseases
ROS: Reactive oxygen species
LOX: Lipoxygenase
HYAL: Hyaluronidase
XO: Xanthine oxidase
3-NT: 3-Nitrotyrosine
LOOH: Lipid hydroperoxides
TBARS: Thiobarbituric acid-reactive substances
FRAP: Ferric reducing antioxidant power
NEAC: Nonenzymatic antioxidant capacity of plasma
ONOO^–^: Peroxynitrite
PBMCs: Peripheral blood mononuclear cells
AA: Ascorbic acid
TX: (±)-6-Hydroxy-2,2,7,8-tetramethylchroman-2-carboxylic acid (Trolox®)
AAE: Ascorbic acid equivalents
TE: Trolox® equivalents
ECA: (–)-Epicatechin
PB2: Procyanidin B2
CHA: Chlorogenic acid
IQ: Isoquercitrin
RT: Rutin
QU: Quercetin
IND: Indomethacin
TPC: Total phenolic content (Folin-Ciocalteu assay)
GAE: Gallic acid equivalents
TPH: Total phenolic content (HPLC)
TFL: Total flavonoid content
TCFA: Total content of caffeic acid derivatives
TCHA: Total content of CHA isomers
TPA: Total proanthocyanidin content (*n*-butanol/HCl assay)
CYE: Cyanidin chloride equivalents
TLPA: Total content of low-molecular-mass flavanols and proanthocyanidins (HPLC).

## References

[1] Pandey K. B., Rizvi S. I. (2009). Plant polyphenols as dietary antioxidants in human health and disease. *Oxidative Medicine and Cellular Longevity*.

[2] Barrajón-Catalán E., Herranz-lópez M., Joven J., Camps J. (2014). Molecular promiscuity of plant polyphenols in the management of age-related diseases: far beyond their antioxidant properties. *Oxidative Stress and Inflammation in Non-communicable Diseases - Molecular Mechanisms and Perspectives in Therapeutics*.

[3] Biswas S. K. (2016). Does the interdependence between oxidative stress and inflammation explain the antioxidant paradox?. *Oxidative Medicine and Cellular Longevity*.

[4] Hussain T., Tan B., Yin Y., Blachier F., Tossou M. C. B., Rahu N. (2016). Oxidative stress and inflammation: what polyphenols can do for us?. *Oxidative Medicine and Cellular Longevity*.

[5] Franz C., Chizzola R., Novak J., Sponza S. (2011). Botanical species being used for manufacturing plant food supplements (PFS) and related products in the EU member states and selected third countries. *Food and Function*.

[6] McAllister H. A. (2005). *The Genus Sorbus: Mountain Ash and Other Rowans*.

[7] Kültür S. (2007). Medicinal plants used in Kirklareli Province (Turkey). *Journal of Ethnopharmacology*.

[8] Shikov A. N., Pozharitskaya O. N., Makarov V. G., Wagner H., Verpoorte R., Heinrich M. (2014). Medicinal plants of the Russian Pharmacopoeia; their history and applications. *Journal of Ethnopharmacology*.

[9] Kylli P., Nohynek L., Puupponen-Pimiä R. (2010). Rowanberry phenolics: compositional analysis and bioactivities. *Journal of Agricultural and Food Chemistry*.

[10] Olszewska M. A., Presler A., Michel P. (2012). Profiling of phenolic compounds and antioxidant activity of dry extracts from the selected Sorbus species. *Molecules*.

[11] Raudonė L., Raudonis R., Gaivelytė K. (2015). Phytochemical and antioxidant profiles of leaves from different *Sorbus* L. species. *Natural Product Research*.

[12] Hukkanen A. T., Pölönen S. S., Kärenlampi S. O., Kokko H. I. (2006). Antioxidant capacity and phenolic content of sweet rowanberries. *Journal of Agricultural and Food Chemistry*.

[13] Olszewska M. A., Michel P. (2009). Antioxidant activity of inflorescences, leaves and fruits of three *Sorbus* species in relation to their polyphenolic composition. *Natural Product Research*.

[14] Olszewska M. A., Nowak S., Michel P., Banaszczak P., Kicel A. (2010). Assessment of the content of phenolics and antioxidant action of inflorescences and leaves of selected species from the genus *Sorbus* sensu stricto. *Molecules*.

[15] Ozdal T., Capanoglu E., Altay F. (2013). A review on protein-phenolic interactions and associated changes. *Food Research International*.

[16] Eghbaliferiz S., Iranshahi M. (2016). Prooxidant activity of polyphenols, flavonoids, anthocyanins and carotenoids: updated review of mechanisms and catalyzing metals. *Phytotherapy Research*.

[17] Michel P., Dobrowolska A., Kicel A. (2014). Polyphenolic profile, antioxidant and anti-inflammatory activity of eastern teaberry (*Gaultheria procumbens* L.) leaf extracts. *Molecules*.

[18] Fu R., Zhang Y., Guo Y., Liu F., Chen F. (2014). Determination of phenolic contents and antioxidant activities of extracts of *Jatropha curcas* L. seed shell, a by-product, a new source of natural antioxidant. *Industrial Crops and Products*.

[19] Czerwińska M., Kiss A. K., Naruszewicz M. (2012). A comparison of antioxidant activities of oleuropein and its dialdehydic derivative from olive oil, oleacein. *Food Chemistry*.

[20] Banothu V., Neelagiri C., Adepally U., Lingam J., Bommareddy K. (2017). Phytochemical screening and evaluation of *in vitro* antioxidant and antimicrobial activities of the indigenous medicinal plant *Albizia odoratissima*. *Pharmaceutical Biology*.

[21] Krzyzanowska-Kowalczyk J., Kolodziejczyk-Czepas J., Kowalczyk M., Pecio Ł., Nowak P., Stochmal A. (2017). Yunnaneic acid B, a component of *Pulmonaria officinalis* extract, prevents peroxynitrite-induced oxidative stress in vitro. *Journal of Agricultural and Food Chemistry*.

[22] Kolodziejczyk-Czepas J., Wachowicz B., Moniuszko-Szajwaj B., Kowalska I., Oleszek W., Stochmal A. (2013). Antioxidative effects of extracts from *Trifolium* species on blood platelets exposed to oxidative stress. *Journal of Physiology and Biochemistry*.

[23] Kolodziejczyk J., Olas B., Wachowicz B., Szajwaj B., Stochmal A., Oleszek W. (2011). Clovamide-rich extract from *Trifolium pallidum* reduces oxidative stress-induced damage to blood platelets and plasma. *Journal of Physiology and Biochemistry*.

[24] Kolodziejczyk-Czepas J., Nowak P., Kowalska I., Stochmal A. (2014). Biological activity of clovers-free radical scavenging ability and antioxidant action of six *Trifolium* species. *Pharmaceutical Biology*.

[25] Matczak M., Marchelak A., Michel P. (2018). *Sorbus domestica* L. leaf extracts as functional products: phytochemical profiling, cellular safety, pro-inflammatory enzymes inhibition and protective effects against oxidative stress *in vitro*. *Journal of Functional Foods*.

[26] Clifford M. N., Johnston K. L., Knight S., Kuhnert N. (2003). Hierarchical scheme for LC-MS*^n^* identification of chlorogenic acids. *Journal of Agricultural and Food Chemistry*.

[27] Clifford M. N., Knight S., Kuhnert N. (2005). Discriminating between the six isomers of dicaffeoylquinic acid by LC-MS*^n^*. *Journal of Agricultural and Food Chemistry*.

[28] Cuyckens F., Claeys M. (2004). Mass spectrometry in the structural analysis of flavonoids. *Journal of Mass Spectrometry*.

[29] Hamed A. I., Al-Ayed A. S., Moldoch J., Piacente S., Oleszek W., Stochmal A. (2014). Profiles analysis of proanthocyanidins in the argun nut (*Medemia argun* - an ancient Egyptian palm) by LC-ESI-MS/MS. *Journal of Mass Spectrometry*.

[30] Jaiswal R., Sovdat T., Vivan F., Kuhnert N. (2010). Profiling and characterization by LC-MS*^n^* of the chlorogenic acids and hydroxycinnamoylshikimate esters in maté (*Ilex paraguariensis*). *Journal of Agricultural and Food Chemistry*.

[31] Vukics V., Guttman A. (2010). Structural characterization of flavonoid glycosides by multi-stage mass spectrometry. *Mass Spectrometry Reviews*.

[32] Zhang L., Tu Z. C., Xie X. (2016). Antihyperglycemic, antioxidant activities of two *Acer palmatum* cultivars, and identification of phenolics profile by UPLC-QTOF-MS/MS: new natural sources of functional constituents. *Industrial Crops and Products*.

[33] Elejalde-Palmett C., de Bernonville T. D., Glevarec G. (2015). Characterization of a spermidine hydroxycinnamoyltransferase in *Malus domestica* highlights the evolutionary conservation of trihydroxycinnamoyl spermidines in pollen coat of core eudicotyledons. *Journal of Experimental Botany*.

[34] Olszewska M. A., Roj J. M. (2011). Phenolic constituents of the inflorescences of *Sorbus torminalis* (L.) Crantz. *Phytochemistry Letters*.

[35] Olszewska M. A., Michel P. (2012). Activity-guided isolation and identification of free radical-scavenging components from various leaf extracts of *Sorbus aria* (L.) Crantz. *Natural Product Research*.

[36] Dauguet J. C., Bert M., Dolley J., Bekaert A., Lewin G. (1993). 8-Methoxykaempferol 3-neohesperidoside and other flavonoids from bee pollen of *Crataegus monogyna*. *Phytochemistry*.

[37] Tang W., Hioki H., Harada K., Kubo M., Fukuyama Y. (2007). Antioxidant phenylpropanoid-substituted epicatechins from *Trichilia catigua*. *Journal of Natural Products*.

[38] Grzesik M., Naparło K., Bartosz G., Sadowska-Bartosz I. (2018). Antioxidant properties of catechins: comparison with other antioxidants. *Food Chemistry*.

[39] Perez-Vizcaino F., Duarte J. (2010). Flavonols and cardiovascular disease. *Molecular Aspects of Medicine*.

[40] Valentová K., Vrba J., Bancířová M., Ulrichová J., Křen V. (2014). Isoquercitrin: pharmacology, toxicology, and metabolism. *Food and Chemical Toxicology*.

[41] Manach C., Williamson G., Morand C., Scalbert A., Rémésy C. (2005). Bioavailability and bioefficacy of polyphenols in humans. I. Review of 97 bioavailability studies. *The American Journal of Clinical Nutrition*.

[42] Cai H., Boocock D. J., Steward W. P., Gescher A. J. (2007). Tissue distribution in mice and metabolism in murine and human liver of apigenin and tricin, flavones with putative cancer chemopreventive properties. *Cancer Chemotherapy and Pharmacology*.

[43] Farah A., Monteiro M., Donangelo C. M., Lafay S. (2008). Chlorogenic acids from green coffee extract are highly bioavailable in humans. *Journal of Nutrition*.

[44] Gil M., Wianowska D. (2017). Chlorogenic acids – their properties, occurrence and analysis. *Annales Universitatis Mariae Curie-Sklodowska Lublin-Polonica*.

[45] Williamson G., Manach C. (2005). Bioavailability and bioefficacy of polyphenols in humans. II. Review of 93 intervention studies. *The American Journal of Clinical Nutrition*.

[46] Marengo B., Nitti M., Furfaro A. L. (2016). Redox homeostasis and cellular antioxidant systems: crucial players in cancer growth and therapy. *Oxidative Medicine and Cellular Longevity*.

[47] Szabó C., Ischiropoulos H., Radi R. (2007). Peroxynitrite: biochemistry, pathophysiology and development of therapeutics. *Nature Reviews Drug Discovery*.

[48] Cheng Y.-C., Sheen J.-M., Hu W. L., Hung Y.-C. (2017). Polyphenols and oxidative stress in atherosclerosis-related ischemic heart disease and stroke. *Oxidative Medicine and Cellular Longevity*.

[49] Pacher P., Beckman J. S., Liaudet L. (2007). Nitric oxide and peroxynitrite in health and disease. *Physiological Reviews*.

[50] Marrocco I., Altieri F., Peluso I. (2017). Measurement and clinical significance of biomarkers of oxidative stress in humans. *Oxidative Medicine and Cellular Longevity*.

[51] Frijhoff J., Winyard P. G., Zarkovic N. (2015). Clinical relevance of biomarkers of oxidative stress. *Antioxidants & Redox Signaling*.

[52] Miao J.-L., Wang W.-F., Pan J.-X., Lu C.-Y., Li R.-Q., Yao S.-D. (2001). The scavenging reactions of nitrogen dioxide radical and carbonate radical by tea polyphenol derivatives: a pulse radiolysis study. *Radiation Physics and Chemistry*.

[53] German Nutrition Society (DGE) (2015). New reference values for vitamin C intake. *Annals of Nutrition and Metabolism*.

[54] Deicher R., Ziai F., Bieglmayer C., Schillinger M., Hörl W. H. (2005). Low total vitamin C plasma level is a risk factor for cardiovascular morbidity and mortality in hemodialysis patients. *Journal of the American Society of Nephrology*.

[55] Girish K. S., Kemparaju K., Nagaraju S., Vishwanath B. S. (2009). Hyaluronidase inhibitors: a biological and therapeutic perspective. *Current Medicinal Chemistry*.

[56] Schneider I., Bucar F. (2005). Lipoxygenase inhibitors from natural plant sources. Part 2: medicinal plants with inhibitory activity on arachidonate 12-lipoxygenase, 15-lipoxygenase and leukotriene receptor antagonists. *Phytotherapy Research*.

[57] Pacher P., Nivorozhkin A., Szabó C. (2006). Therapeutic effects of xanthine oxidase inhibitors: renaissance half a century after the discovery of allopurinol. *Pharmacological Reviews*.

